# circCPA4 induces malignant behaviors of prostate cancer via miR-491-5p/SHOC2 feedback loop

**DOI:** 10.1016/j.clinsp.2023.100314

**Published:** 2024-01-13

**Authors:** Wenqing Xu, Zhihong Zhong, Long Gu, Yiming Xiao, BinShen Chen, Weilie Hu

**Affiliations:** a Department of Urology, The First School of Clinical Medicine of Southern Medical University, Guangzhou City, Guangdong Province, China; b Department of Urology, Guangzhou Development District Hospital, Guangzhou City, Guangdong Province, China; c Department of Urology, Zhujiang Hospital of Southern Medical University, Guangzhou City, Guangdong Province, China

**Keywords:** circCPA4, Prostate cancer, miR-491-5p, SHOC2, Invasion

## Abstract

**Objective:**

circCPA4 has been defined to be an oncogenic gene. This study examined whether circCPA4 regulates Prostate Cancer (PC) development and revealed its molecular mechanism.

**Methods:**

PC tissues and PC cell lines were collected, in which circCPA4/miR-491-5p/SHOC2 levels were evaluated by RT-qPCR and immunoblot. Colony formation assay and EdU assay assessed cell proliferation, flow cytometry measured apoptosis, and Transwell assessed invasion and migration. Ki-67, cleaved caspase-3, E-cadherin, and N-cadherin were evaluated by immunoblot. Based on the luciferase reporter assay and RIP assay the authors investigated the targeting relationship between circCPA4/miR-491-5p/SHOC2. The effect of circCPA4 on tumor growth was evaluated by xenotransplantation in nude mice.

**Results:**

circCPA4 and SHOC2 levels were abundant while miR-491-5p expression was low in PC. Loss of circCPA4 decreased the proliferation and EdU-positive rate of PC cells, enhanced apoptosis, and inhibited invasion, migration, and EMT. Upregulation of circCPA4 forced the malignant behaviors of PC cells, and this promotion could be abolished when miR-491-5p was overexpressed or SHOC2 was silenced. CircCAP4 competitively decoyed miR-491-5p mediating SHOC2 expression. circCAP4 suppression inhibited PC tumor growth.

**Conclusion:**

circCAP4 acts as a novel oncogenic factor in PC, accelerating the malignant behavior of PC cells via miR-491-5p/SHOC2 interaction. This novel ceRNA axis may be a potential target for PC drug development and targeted therapy in the future.

## Highlights

• circCPA4 deficiency inhibits PC3 cell metastasis.
• circCPA4 interacts with miR-491-5p in affecting the biological behavior of PC3 cells.
• circCPA4 mediates and affects the biological behavior of PC3.

## Introduction

Prostate Cancer (PC) is a common cancer type affecting men's health worldwide.^1^ PC originates from the gland epithelial cells of the prostate and is characterized by the uncontrolled growth and proliferation of cancer cells.^2^ Currently, treatment options for PC include surgery, immunotherapy, hormone therapy, radiation therapy, and chemotherapy.^3^^,^^4^ Despite advances in PC treatment, mortality and morbidity associated with the disease remain high, and new treatment strategies are urgently needed.

Upon reverse splicing, circRNAs are formed by covalently linking their downstream 3′end and upstream 5′end.^5^ circRNAs are expressed in large quantities in tissues and involve gene regulation, including transcription and post-transcriptional regulation.^6^ Dysregulation of circRNAs in cancers, including PC, has been shown, indicating their potential as a diagnostic and therapeutic target.^7^^,^^8^ circCPA4 has been elucidated to be abundant in cancers and involved in the biological activities of cancer cells.^9^, ^10^, ^11^ However, its biological function in PC remains unclear. miRNAs are crucial in gene expression modification^12^ and miRNAs can target specific mRNAs and regulate their expression, while circRNAs can act as a sponge for miRNAs and regulate their availability to targeted mRNAs.^13^ This means that miRNA and circRNA can interact with each other to form complex regulatory networks and participate in the regulation of various activities of PC.^14^ However, whether circCPA4 regulates miRNA/mRNA networks and is involved in PC development remains unclear.

This study investigated the biological function of circCPA4 in PC and put forward a hypothesis that circCPA4 participates in the development process of PC by regulating downstream miRNA/mRNA networks.

## Materials and methods

### Clinical samples

This study obtained approval by the Ethics Committee of The First School of Clinical Medicine of Southern Medical University. All patients presented informed consent. Thirty-nine pairs of PC tissue samples and normal tissue samples were obtained from The First School of Clinical Medicine of Southern Medical University and confirmed by the pathologist. Patients with preoperative pathological diagnosis of PC signed informed consent before being enrolled in the study. Patients were excluded if they had a second primary tumor, HIV or syphilis virus, severe liver, kidney or other systemic disease, or other malignant disease, and had received chemotherapy or radiation before surgery. After excision or biopsy, the tissue was rapidly frozen and stored at -80°C.

### RT-qPCR

A NanoDrop 2000 spectrophotometer (Thermo Fisher Scientific) measured the quality of RNA obtained from cells/tissues using a Trizol reagent (Life Technologies). PrimeScript RT Master Mix (RR036A; Takara) was employed to generate cDNA of circRNA and mRNA, whereas PrimeScript™RT Reagent Kit (RR037A; Takara) was purchased for miRNA reverse transcription. ChamQ SYBR qPCR Master Mix (Q311-02; Vazyme) and Roter Gene 3000 sequence detection system (Corbett Research, Australia) performed PCR. To calculate relative gene expression, 2^−ΔΔCt^ method was utilized, and GAPDH or U6 was considered endogenous control. The primer sequence is found in Table 1.

**Table 1:** PCR primer sequences.

	**Primer sequences (5′ - 3′)**
**circCPA4**	Forward: 5′- TCAAACTCAAGCCCTTTTAGACA-3′
	Reserse: 5′- TCAGGAAAGTCTGCGGCAAT-3′
**miR-491-5p**	Forward: 5′-GAGTGGGGAACCCTTCC-3′
	Reserse: 5′- TGGTGTCGTGGAGTCG-3′
**SHOC2**	Forward: 5′- CCAGCAGAGGTGGGATGTTT-3′
	Reserse: 5′- GTGCTGCAAGTCAAGGTTGG-3′
**U6**	Forward: 5′- CTCGCTTCGGCAGCACA-3′
	Reserse: 5′- AACGCTTCACGAATTTGCGT-3′
**GAPDH**	Forward: 5′- CACCCACTCCTCCACCTTTG-3′
	Reserse: 5′- CCACCACCCTGTTGCTGTAG-3′

### Cell culture

PC cell lines (PC3, DU145, VCaP, and 22RV1) and normal prostate cell lines (RWPE) were derived from ATCC. For the 4 PC cell lines, the cell culture was RPMI-1640 (Gibco) plus 10 % FBS (Gibco), 100 U/mL penicillin and 100 μg/mL streptomycin (Invitrogen). Keratinocyte serum-free medium (Gibco) was adopted to culture RWPE cells. A humidified atmosphere was needed for cell culture at 37°C and 5 % CO_2_.

### Actinomycin D treatment

PC3 cells were plated in 6-well plates (5×10^5^ cells/well) and allowed to 2h culture. RNA stability was analyzed by PCR after actinomycin D was administered (2 μg/mL, Sigma) at a specific time point.

### RNAse R treatment

10 μg RNA from PC3 cells was mixed with RNAse R (3 U/g, Epicenter) at 37°C and after 30 min, circRNA and linear RNA were evaluated using PCR.

### FISH assay

RiboBio (Guangzhou, China) provided Cy3-labeled circCPA4 and DIG-labeled lock-in nucleic acid miR-491-5p probes, as well as a FISH kit to obtain images. A1Si laser scanning confocal microscope (Nikon) and ModFit LT software were auxiliary for data analysis.

### Cell transfection

Lipofectamine 3000 (Invitrogen) transfected circCPA4 and SHOC2 targeted siRNA and pcDNA 3.1 overexpression vectors as well as miR-491-5p mimic or miR-491-5p inhibitor into PC3 cells. All vectors were provided by RiboBio. RT-qPCR performed after 48 transfections verified the transfection efficiency.

### Colony formation test

Six-well plates were covered with RPMI-1640 containing 10 % FBS, in which cells were seeded at 2000 cells/well for 10d. After fixation and crystal violet staining, the colonies were imaged and counted.

### EdU experiment

EdU experiment was conducted using the cell-light EdU Apollo 488 In Vitro kit (C10310-3; RiboBio). In brief, cells were fixed with 4 % paraformaldehyde (P0099; Beyotime) and permeated with 0.5 % Triton X-100 after EdU staining (50 nM) and stained with 1×Apollo® fluorescent dye. The nuclei were restained with DAPI. EdU-positive cells were photographed using FSX100 microscope (Olympus).

### Flow cytometry

PC3 cells were assayed by Annexin V-FITC apoptosis detection kit (Vazyme). Cells after rinsing with cold PBS (Sangon, China) were resuspended in a binding buffer and reacted darkly with 5 µL Annexin V-FITC and 5 µL PI for 15 min. Analyses of flow cytometry (Beckman Coulter, USA) were conducted to determine apoptosis rates.

### Transwell tests

The Transwell chamber (Corning, USA) was equipped with an 8 μm pore size polycarbonate filter. Cells (5×10^4^) in serum-free medium were coated on the upper cavity, in which Matrigel (BD356230, Corning) was only required for invasion assay. Meanwhile, 600 μL 10 % FBS was fulfilled with the lower cavity. After culture, cells transferred to the lower cavity were fixed in 95 % ethanol for 15 min, stained with 0.1 % crystal violet for 20 min, and counted under a microscope (Leica Microsystems) in 5 regions.

### Immunoblot

Proteins were isolated from tissues and cells using RIPA lysis buffer (Thermo Fisher) and quantified using a BCA analysis kit (BioVision, USA). The proteins were separated on a 10 % SDS-polyacrylamide gel and transferred to a PVDF membrane (Thermo Fisher) which was covered with 5 % milk and incubated with the primary antibody overnight and the secondary antibody conjugated with horseradish peroxidase (BD Biosciences) for 1h. Protein bands were evaluated using the SynGene system and GeneSnap software (SynGene, USA). Primary antibodies: N-cadherin (13116, Cell Signaling Technology), GAPDH (2118, Cell Signaling Technology), E-cadherin (3195, Cell Signaling Technology), cleaved caspase-3 (9664, Cell Signaling Technology), Ki-67 (ab16667, Abcam).

### Dual luciferase reporter experiment

circCPA4 and SHOC2 Wild Type (WT) sequences containing predicted miR-491-5p binding sites were cloned and inserted into firefly luciferase PCL-3 vectors (E1751, Promega, USA), named circCPA4-WT and SHOC2-WT. circCPA4 and SHOC2 Mutants (MUTs) were inserted into luciferase vectors and named circCPA4-MUT and SHOC2-MUT. For co-transfection of luciferase reporter and miR-491-5p mimic and mimic NC, Lipofectamine 3000 was utilized. Luciferase Reporter Assay System (E1960, Promega) analyzed luciferase activity at 48h.

### RIP experiment

RIP assay was performed using the Magna RIP kit (Millipore). Cells were lysed in 100 μL RIP lysis buffer containing a mixture of RNAse inhibitors (Promega) and protease inhibitors (Roche). After DNase I (Roche) treatment, 900 μL RIP immunoprecipitation buffer was added to the lysate and reacted with antibody-conjugated magnetic beads (anti-AgO2 or anti-IgG). The beads after RIP washing buffer treatment were processed with protease K for 30 min before PCR analysis.

### Tumor growth in vivo

All experimental mouse procedures were approved by the Animal Ethics Committee of The First School of Clinical Medicine of Southern Medical University, and the researchers minimized the suffering of the mice. Six eight-week-old male BALB/c nude mice were purchased from Hunan SJA Animal Experimental Co., Ltd. As for *in vivo* tumor metastasis studies, PC3 cells (5×10^6^ cells) with stable circCPA4 knockdown or non-knockdown circCPA4 were inoculated subcutaneously into mouse armpits. During the following 30d, using a digital caliper, the size of tumors was measured periodically in the mice. Tumor volume (cm^3^) = 0.5 × length × width^2^. After that, the excised tumor was weighed, fixed with 4 % paraformaldehyde, and paraffin-embedded for IHC staining.^15^

### Data analysis

GraphPad Prism 9.0 was utilized for data analysis. Data were presented as mean ± standard deviation. Two-tailed Student *t*-test and one-way ANOVA compared the data. Chi-Square assay assessed the association of circCPA4 with clinicopathologic features in PC patients; *p < 0.05 was statistically significant.

## Results

### circCPA4 high expression in PC

**Fig. 1:**
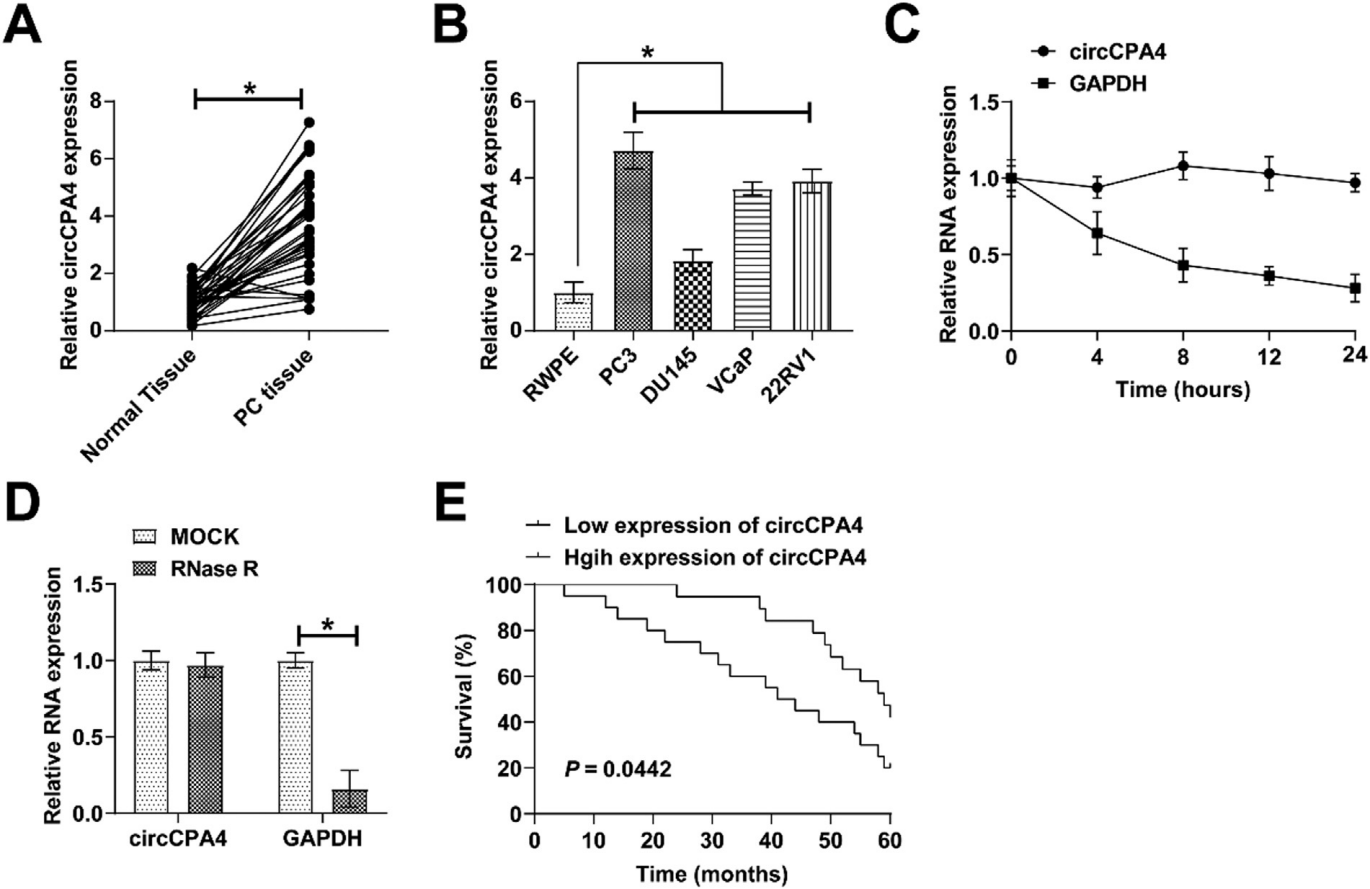
**circCPA4 high expression in PC.** (A‒B) Higher circCPA4 expression in PC tissues versus normal tissues and in PC cell lines versus RWPE. (C‒D) circCPA4 had ring structure and high stability. (E) Negative relationship between circCPA4 and survival prognosis of PC patients.

circCPA4 expression pattern in PC was examined. PC tissues (Fig. 1A) as well as four PC cell lines (Fig. 1B) had higher circCPA4 levels than controls. To examine the ring structure of circCPA4, both actinomycin D and RNAse R treatments were conducted, indicating that the two methods caused no change in the RNA stability of circCPA4, but reduced that of linear RNA (Fig. 1 C and D). Also, circCPA4 could be amplified by outward-diverging primers in cDNA, but circCPA4 could not be amplified from gDNA (Fig. 1E). Abnormal expression of circCPA4 was highly correlated with TNM staging and distal metastasis in PC patients (Table 2). A poorer survival prognosis was observed for those with high circCPA4 expression (Fig. 1F).

**Table 2:** Correlation analysis of clinicopathological characteristics of patients with circCPA4 and PC.

**Characteristic**	**Cases**	**The expression of circCAP4**		**p**
	**n = 39**	**Low (n = 20)**	**High (n = 19)**	
Age (year)				0.3333
≥ 60	28	13	15	
< 60	11	7	4	
Tumor size (cm)				0.4075
≥ 3	17	10	7	
< 3	22	10	12	
Lymph node metastasis				0.2651
Negative	24	14	10	
Positive	15	6	9	
TNM stage				0.0015*
I‒II	26	18	8	
II‒IV	13	2	11	
Distal transfer				0.0008*
Negative	21	16	5	
Positive	18	4	14	

### circCPA4 deficiency inhibits PC3 cell metastasis

**Fig. 2:**
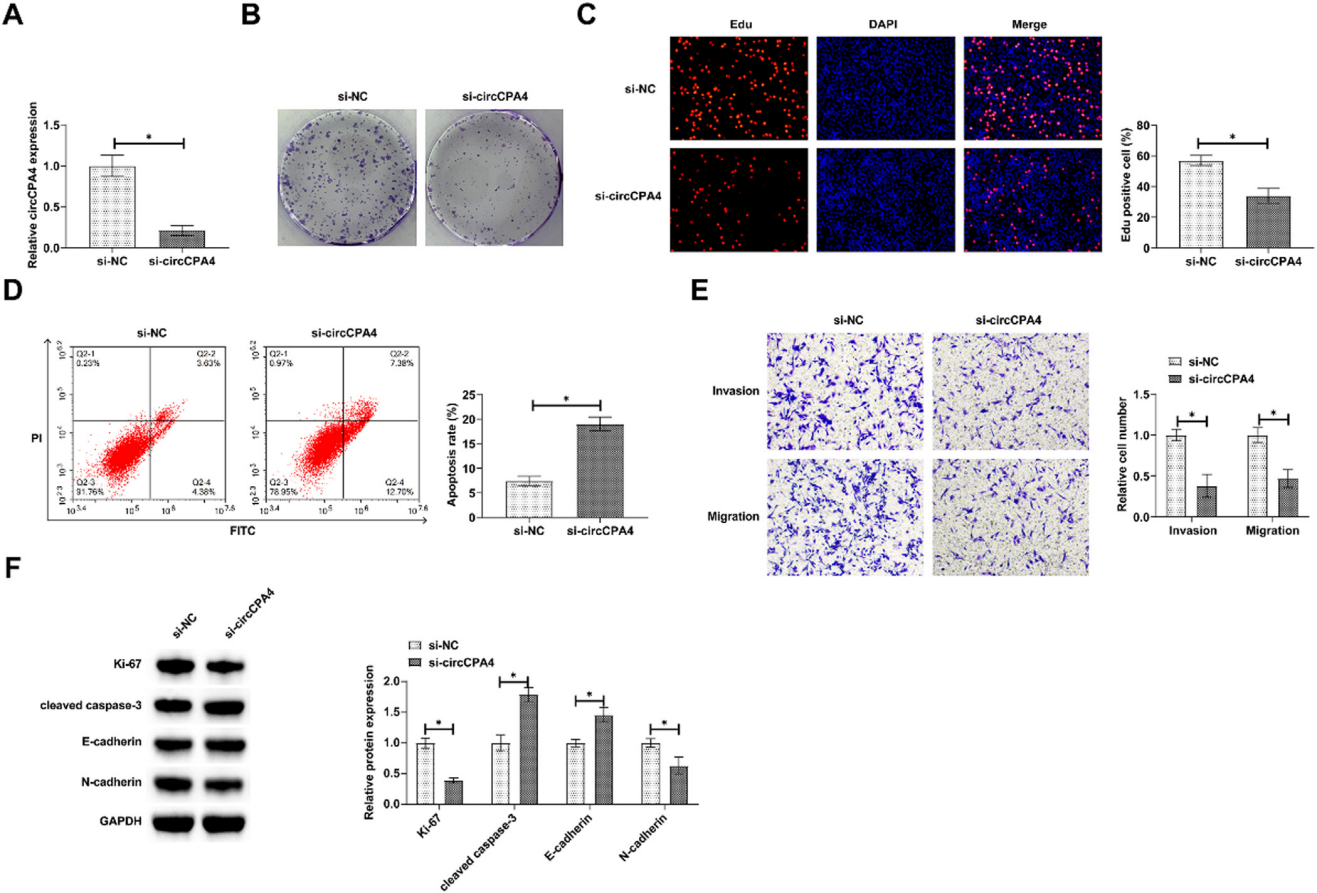
**circCPA4 knockdown suppresses PC3 cell activities.** (A) si-circCPA4 lowered circCPA4 expression. (B‒C) circCPA4 reduced cell proliferation. (D) circCPA4 enhanced apoptosis rate. (E) circCPA4 reduced invasion and migration abilities. (F) circCPA4 reduced Ki-67 and N-cadherin and elevated cleaved caspase-3 and E-cadherin.

circCPA4-targeted siRNA was transfected into PC3 cells to analyze the biological function of circCPA4. Transfection of si-circCPA4 reduced circCPA4 expression effectively (Fig. 2A). Colony formation and EdU tests noted that circCPA4 knockdown reduced cell proliferation capacity and EdU-positive rate (Fig. 2 B and C). Also, circCPA4 depletion inhibited protein expression of proliferating protein Ki-67 (Fig. 2F). Cellular apoptosis was evaluated, as manifested by increased apoptosis, and cleaved caspase-3 protein expression in circCPA4-knockdown cells (Fig. 2 D and F). Transwell assay presented that circCPA4 depletion reduced the number of invading and migrating cells (Fig. 2E) and suppressed N-cadherin and increased E-cadherin expressions (Fig. 2F).

### circCPA4 competitively decoys miR-491-5p

**Fig. 3:**
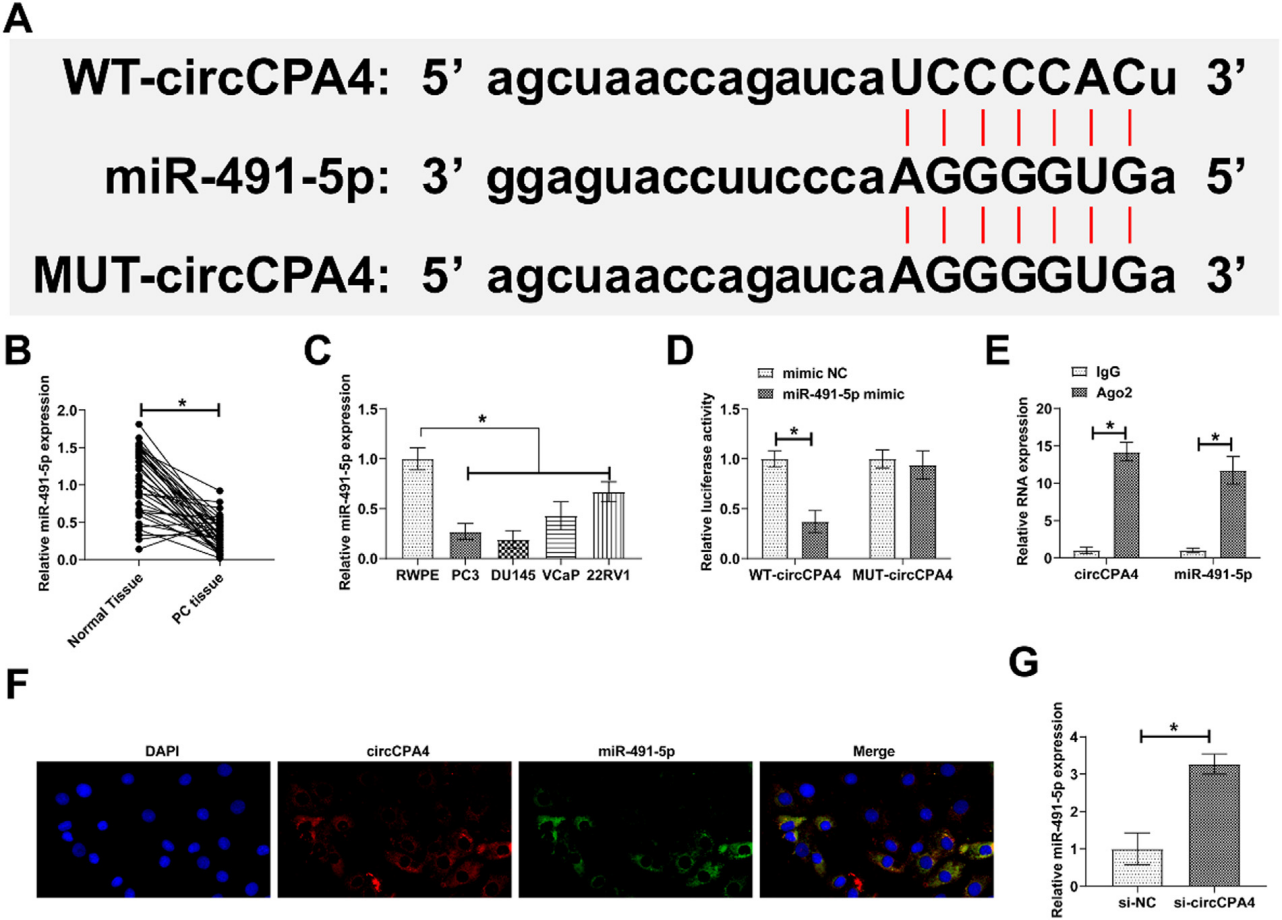
**miR-491-5p is absorbed by circCPA4.** (A) circCPA4 and miR-491-5p had potential binding sites. (B‒C) Lower miR-491-5p expression in PC tissues versus normal tissues and in PC cell lines versus RWPE. (D‒E) circCPA4 and miR-491-5p had a targeting binding relationship. (F) circCPA4 and miR-491-5p were localized in the cytoplasm of PC3 cells. (G) circCPA4 knockdown elevated miR-491-5p expression.

The study predicted from the bioinformatics website that potential binding sites for circCPA4 and miR-491-5p existed (Fig. 3A). miR-491-5p in PC was evaluated by RT-qPCR, showing low expression in PC tissues and four PC cell lines (Fig. 3 B and C). Therefore, circCPA4 might be targeted to miR-491-5p, which was verified by dual luciferase reporter assay and RIP assay. As shown in Fig. 3D, E, WT-circCPA4 in combination with miR-491-5p mimic could reduce luciferase activity, and there was a high level of enrichment in Ago2 beads for circCPA4 and miR-491-5p. FISH experiments further confirmed that circCPA4 and miR-491-5p were colocalized in the cytoplasm of PC3 cells (Fig. 3F). miR-491-5p levels in PC3 cells were restored after circCPA4 knockdown (Fig. 3G).

### circCPA4 interacts with miR-491-5p in affecting the biological behavior of PC3 cells

**Fig. 4:**
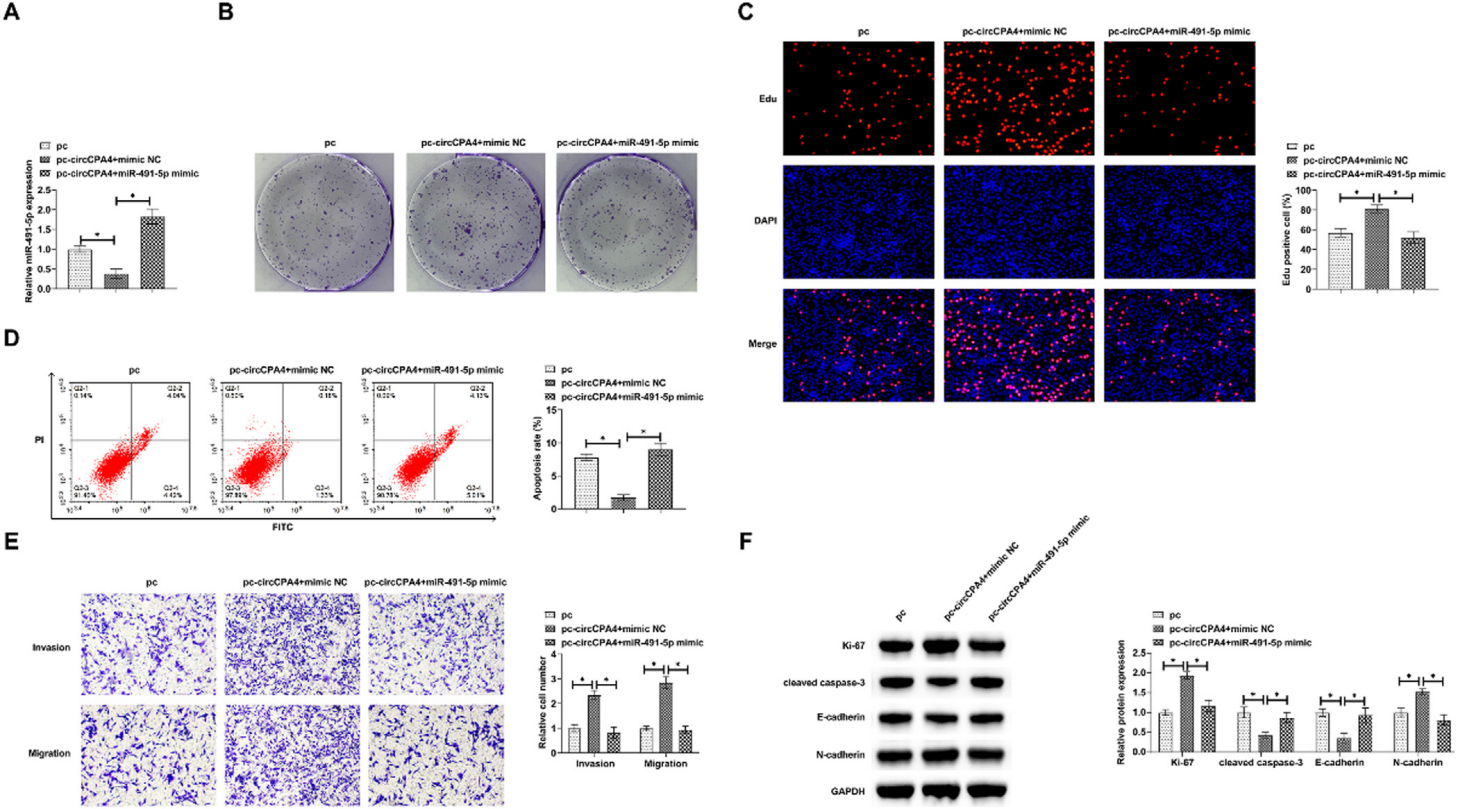
**circCPA4 interacts with miR-491-5p and affects the biological behavior of PC3 cells.** Based on pcDNA 3.1-circCPA4, miR-491-5p mimic was further treated. (A) miR-491-5p mimic restored miR-491-5p expression. (B‒C) miR-491-5p mimic suppressed cell proliferation. (D) miR-491-5p mimic activated apoptosis rate. (E) miR-491-5p mimic suppressed invasion and migration abilities. (F) miR-491-5p mimic suppressed Ki-67 and N-cadherin and enhanced cleaved caspase-3 and E-cadherin.

PC3 cells were co-transfected with pcDNA 3.1-circCPA4 and miR-491-5p mimic. RT-qPCR detected that pcDNA 3.1-circCPA4 promoted circCPA4 and inhibited miR-491-5p levels, while miR-491-5p mimic restored miR-491-5p levels (Fig. 4A). Fig. 4B, 4C, and 4F showed that pcDNA 3.1-circCPA4 increased cell proliferation and EdU positive rate, and promoted Ki-67 expression, but this phenomenon was blocked by miR-491-5p mimic. At the same time, pcDNA 3.1-circCPA4 reduced cell apoptosis rate and inhibited cleaved caspase-3 expression, but this action was impaired by miR-491-5p mimic (Fig. 4 D and F). Transwell tests indicated that pcDNA 3.1-circCPA4 promoted cell invasion and migration ability, but miR-491-5p mimic destroyed this phenomenon (Fig. 4E). Immunoblot suggested that pcDNA 3.1-circCPA4 enhanced N-cadherin and lowered E-cadherin protein expression, but miR-491-5p mimic blocked the changes of these proteins (Fig. 4F).

### miR-491-5p and SHOC2 are interacted

**Fig. 5:**
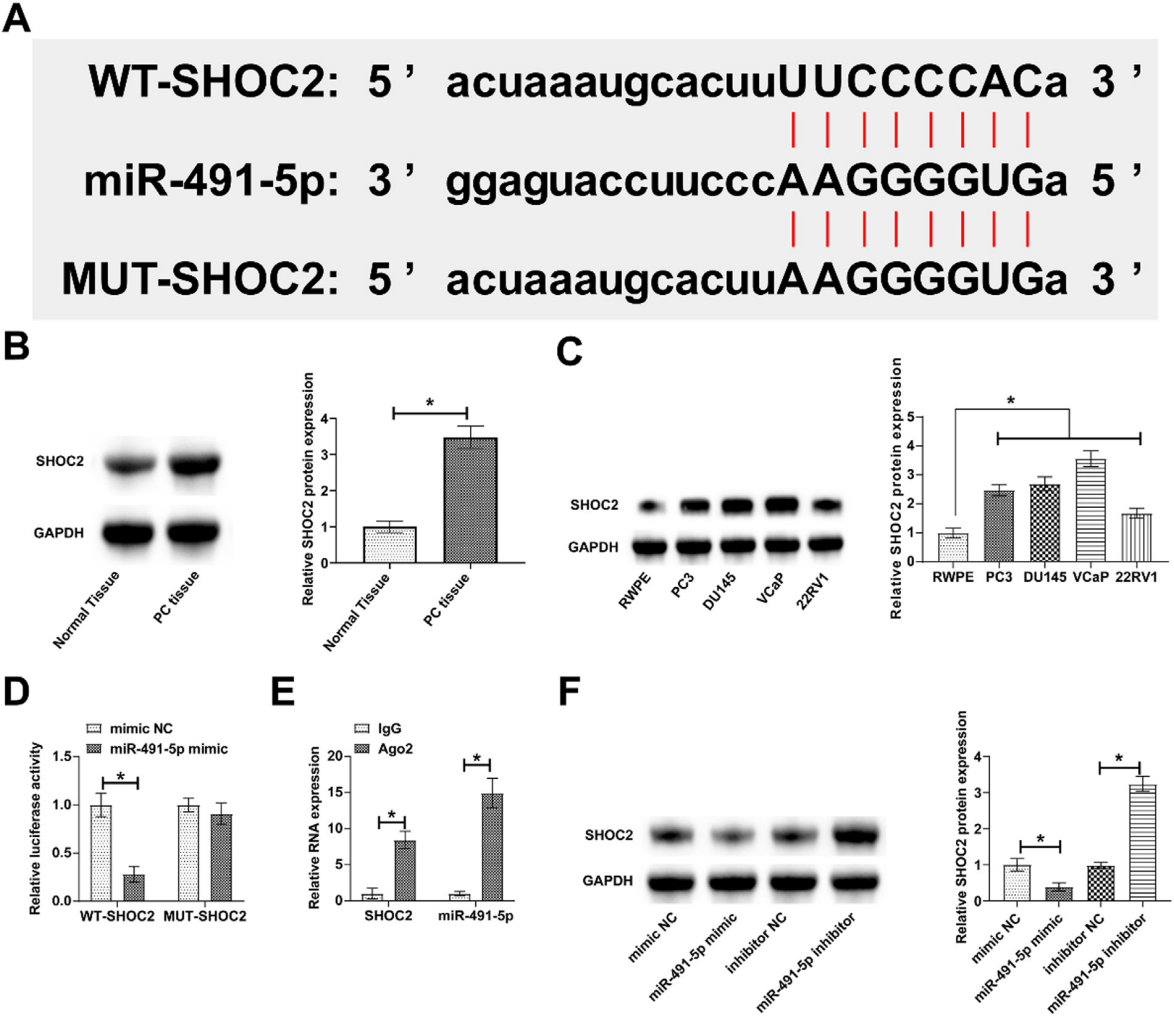
**SHOC2 shows the downstream target gene of miR-491-5p.** (A) SHOC2 and miR-491-5p shared potential binding sites. (B‒C) Higher SHOC2 expression in PC tissues versus normal tissues and in PC cell lines versus RWPE. (D‒E) SHOC2 and miR-491-5p had a targeting binding relationship. (F) miR-491-5p mediated SHOC2 protein expression.

Potential binding sites of miR-491-5p and SHOC2 were predicted through bioinformatics websites (Fig. 5A). SHOC2 expression was then evaluated by immunoblot, presenting a higher level in both PC tissues and cells (Fig. 5 B and C). Dual luciferase reporter experiments addressed that co-transfection with WT-SHOC2 and miR-491-5p mimic reduced luciferase activity (Fig. 5D). RIP experiments observed the enrichment of SHOC2 and miR-491-5p in Ago2 magnetic beads (Fig. 5E). Increasing or decreasing miR-491-5p inhibited and promoted SHOC2 expression in PC3 cells, respectively (Fig. 5F).

### circCPA4 mediates and affects the biological behavior of PC3

**Fig. 6:**
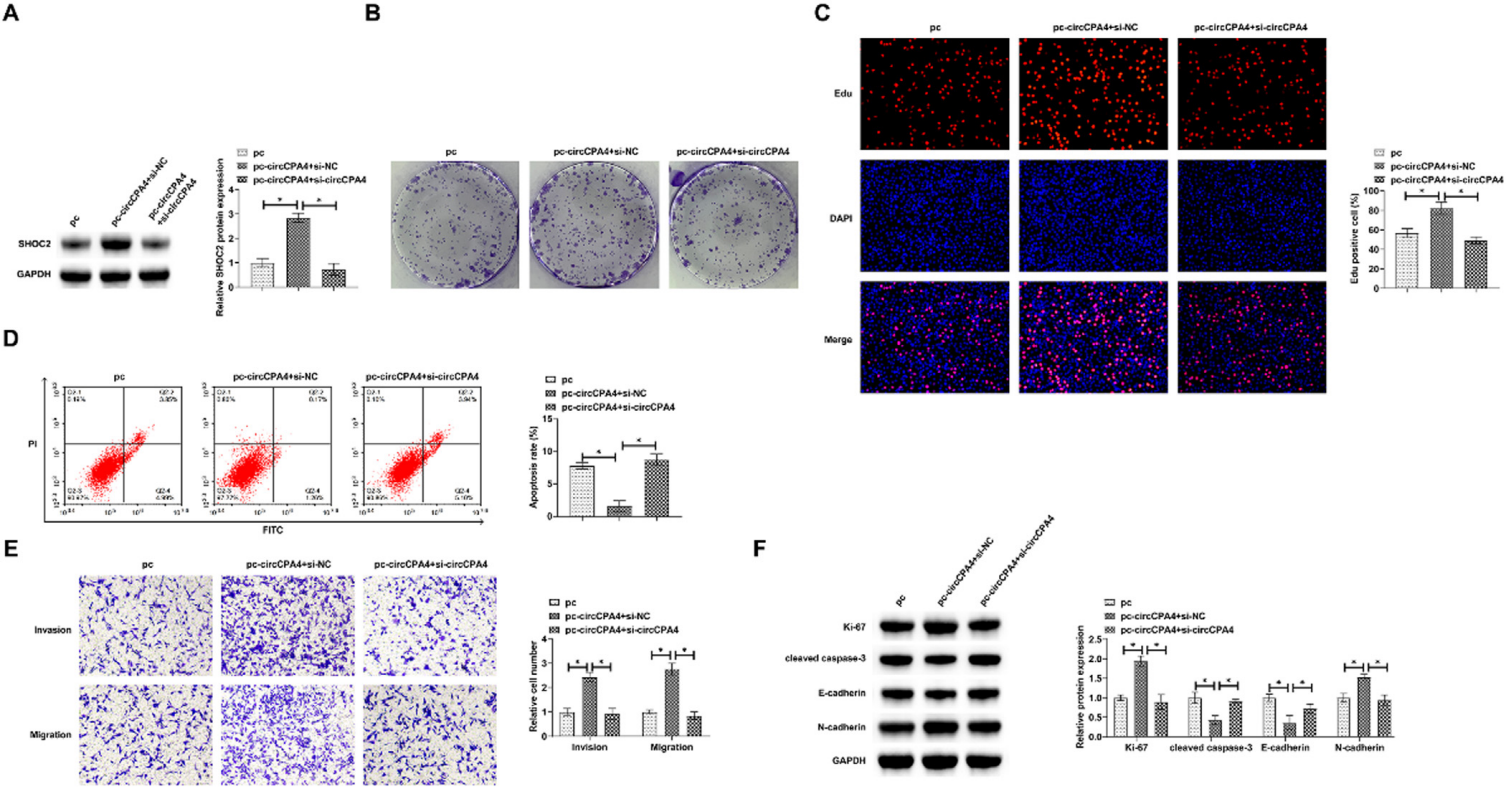
**circCPA4 and SHOC2 affect the biological behavior of PC3 cells.** Based on pcDNA 3.1-circCPA4, si-SHOC2 was further processed. (A) si-SHOC2 lowered SHOC2 expression. (B‒C) si-SHOC2 limited cell proliferation. (D) si-SHOC2 induced apoptosis rate. (E) si-SHOC2 hampered invasion and migration abilities. (F) si-SHOC2 limited Ki-67 and N-cadherin and induced cleaved caspase-3 and E-cadherin.

PC3 cells were co-transfected with pcDNA 3.1-circCPA4 and si-SHOC2. pcDNA 3.1-circCPA4 elevated SHOC2 protein expression, but this effect was suppressed partially by si-SHOC2 (Fig. 6A). pcDNA 3.1-circCPA4 increased cell proliferation, EdU-positive rate, and Ki-67 protein expression, but si-SHOC2 obstructed this phenomenon (Fig. 6 B, C, F). Additionally, pcDNA 3.1-circCPA4 reduced apoptosis and cleaved caspase-3 protein levels, while si-SHOC2 prevented this (Fig. 6 D and F). Transwell manifested that pcDNA 3.1-circCPA4 increased the number of invasive and migratory cells, while si-SHOC2 blocked this effect (Fig. 6E). Immunoblot determined that pcDNA 3.1-circCPA4 promoted N-cadherin expression and decreased E-cadherin expression, while si-SHOC2 prevented alternations in these proteins (Fig. 6F).

### circCPA4 knockdown inhibits PC tumor growth

**Fig. 7:**
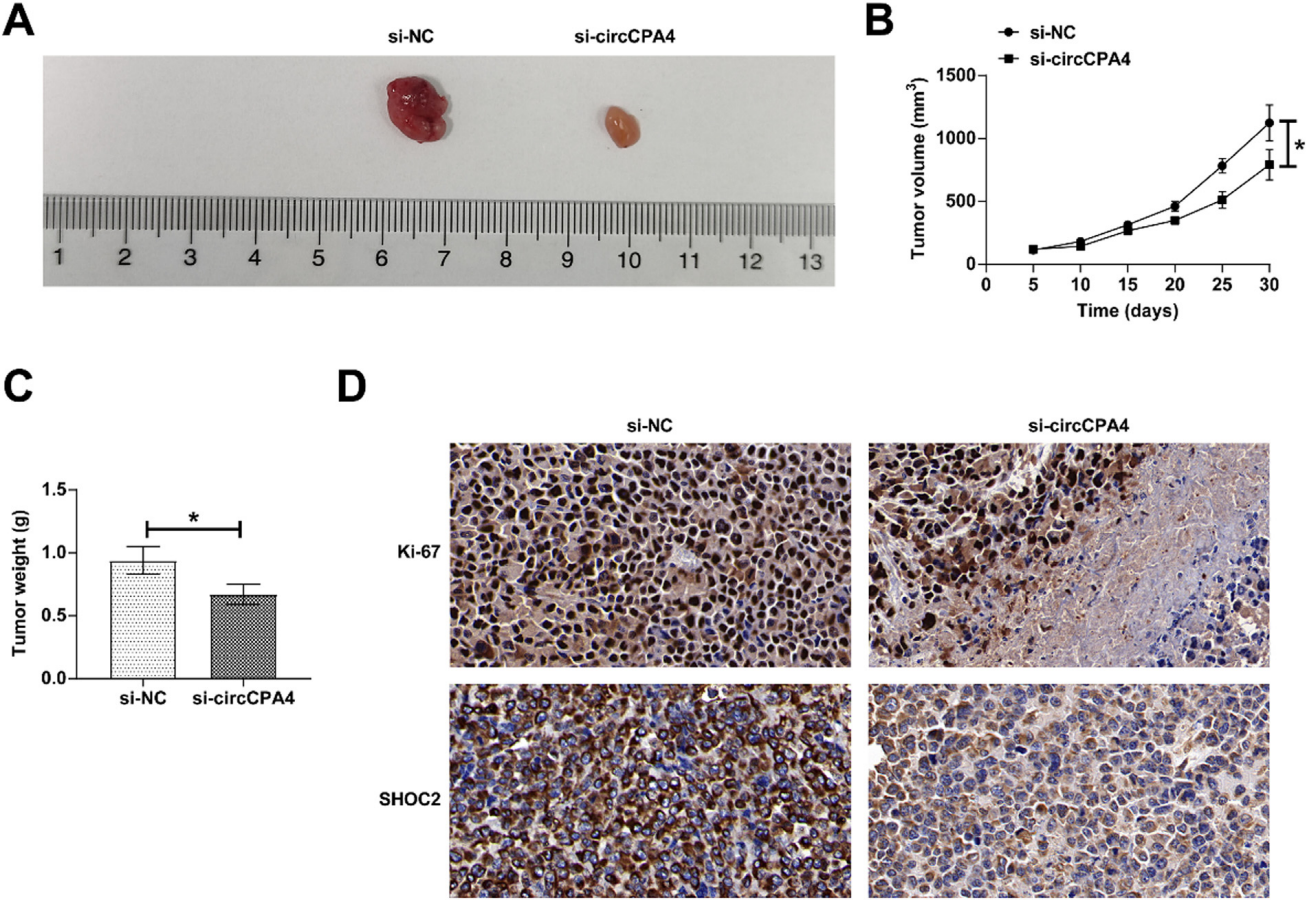
**Inhibition of PC tumor growth by knocking down circCPA4.** (A) Representative pictures of tumors. (B) tumor volume. (C) tumor weight. (D) SHOC2 and Ki-67 expression in tumors (IHC staining). Data were expressed as mean ± SD. *p < 0.05. For Figure 1‒6, n = 3; for Figure 7, n = 5.

Tumor growth was observed after circCPA4 interference in mice. Knocking down circCPA4 lowered tumor volume and weight (Fig. 7 A‒C). IHC staining suggested that circCPA4 deficiency reduced SHOC2 and Ki-67 expressions in tumors (Fig. 7D).

## Discussion

Abnormal expression of circRNA affects PC development.^16^^,^^17^ This study highlights that downregulating circCPA4 inhibited the metastasis of PC. The identification of miR-491-5p/SHOC2 axis as a downstream target of circCPA4 provides a potential pathway for developing targeted therapeutics for PC.

In fact, circCPA4 has been emphasized to correlate with TNM staging, lymph node metastasis, and tumor size in lung cancer patients.^10^ The study further confirmed that abnormally high expression of circCPA4 was associated with TNM staging and distal metastasis in PC patients. In addition, circCPA4 expression levels had prognostic values for overall survival in PC patients. This highlights the potential clinical role of circCPA4 as a diagnostic and prognostic biomarker for PC. Thus, a deeper understanding of the molecular mechanisms of circCPA4′s role in PC could provide new insights into disease pathogenesis.

This study declared that circCPA4 was involved in regulating the proliferation, invasion, and migration, apoptosis, and EMT processes of PC3 cells. EMT is a key process of cancer invasion and metastasis.^18^^,^^19^ Since high circCPA4 is highly associated with remote metastasis in PC patients, it is suggested that circCPA4′s promotion of invasive migration and EMT processes will contribute to remote metastasis of PC. In addition, it has been declared that high expression of circCPA4 can block apoptosis and induce proliferation of cancer,^11^^,^^20^ showing consistency with the results of this study.

Notably, this study further confirmed the ring structure of circCPA4 in PC3 cells. Compared with linear RNA, circRNA is more stable.^21^^,^^22^ Therefore, circCPA4 has a more permanent role in regulating downstream miRNA expression and function, which will continue to influence the malignant development of PC. circCAP4 regulates the biological behavior of different cancers through miRNA/mRNA networks. These miRNA/mRNA networks include miR-214-3p/TGIF2^10^ and miR-760/MEF2D.^9^ This study identified a novel mechanism network for circCPA4 in PC. circCPA4 promoted SHOC2 expression through competitive adsorption of miR-491-5p, thus accelerating multiple biological behaviors of PC3 cells.

However, understanding of the molecular mechanism and biological function of circCPA4 is still limited, and further studies are needed. Secondly, circRNA and miRNA/mRNA targeting regulatory network is extremely complex, which needs to be improved in subsequent studies. In addition, how to detect circCPA4 quickly, effectively, and accurately in the clinic is also a problem that needs to be solved.

Together, the circCPA4/mi-491-5p/SHOC2 axis is involved in PC by regulating cell proliferation, invasion and migration, apoptosis, and EMT. Thus, circCPA4 may be a promising target for developing novel diagnostic and therapeutic strategies for PC.

## Data available

Data is available from the corresponding author on request.

## Ethical approval

All procedures performed in this study involving human participants were in accordance with the ethical standards of the institutional and/or national research committee and with the 1964 Helsinki Declaration and its later amendments or comparable ethical standards. All subjects were approved by The First School of Clinical Medicine of Southern Medical University.

This study followed the Guide for the Care and Use of Laboratory Animals published by the US National Institutes of Health. The animal experiments were approved by the ethics committee of The First School of Clinical Medicine of Southern Medical University.

## Declaration of Competing Interest

The authors declare no conflicts of interest.
